# Risk Factors in and Long-Term Survival of Patients with Post-Transplantation Diabetes Mellitus: A Retrospective Cohort Study

**DOI:** 10.3390/ijerph17124581

**Published:** 2020-06-25

**Authors:** Ching-Yao Cheng, Cheng-Hsu Chen, Ming-Fen Wu, Ming-Ju Wu, Jun-Peng Chen, Ying-Mei Liu, Yu-Chi Hou, Hue-Yu Wang

**Affiliations:** 1 Department of Pharmacy, Taichung Veterans General Hospital, Taichung 40705, Taiwan; chingyao4937b@gmail.com (C.-Y.C.); mfenwu@vghtc.gov.tw (M.-F.W.); lym0361@gmail.com (Y.-M.L.); 2 School of Pharmacy, China Medical University, Taichung 40402, Taiwan; hou5133@gmail.com; 3 Division of Nephrology, Department of Internal Medicine, Taichung Veterans General Hospital, Taichung 40705, Taiwan; cschen@vghtc.gov.tw (C.-H.C.); wmj530@gmail.com (M.-J.W.); 4 Department of Life Science, Tunghai University, Taichung 40704, Taiwan; 5 School of Medicine, College of Medicine, China Medical University, Taichung 40402, Taiwan; 6 School of Medicine, Chung-Shan Medical University, Taichung 40402, Taiwan; 7 Graduate Institute of Clinical Medical Science, College of Medicine, China Medical University, Taichung 40402, Taiwan; 8 Graduate Institute of Biomedical Science, National Chung Hsing University, Taichung 40227, Taiwan; 9 Biostatistics Task Force, Taichung Veterans General Hospital, Taichung 40705, Taiwan; pippan7676@vghtc.gov.tw; 10 Department of Pharmacy, Chi Mei Medical Center, Tainan City 71004, Taiwan; 11 Department of Pharmacy, Chia Nan University of Pharmacy and Science, Tainan City 71710, Taiwan

**Keywords:** immunosuppressant, post-transplant diabetes mellitus, risk factor, survival rate

## Abstract

Post-transplant diabetes mellitus (PTDM) is associated with infection, cardiovascular morbidity, and mortality. A retrospective cohort study involving patients who underwent renal transplantation in a transplantation center in Taiwan from January 2000 to December 2018 was conducted to investigate the incidence and risk factors of PTDM and long-term patient and graft survival rates. High age (45–65 vs. <45 years, adjusted odds ratio (aOR) = 2.90, 95% confidence interval (CI) = 1.64–5.13, *p* < 0.001), high body mass index (>27 vs. <24 kg/m^2^, aOR = 5.35, 95% CI = 2.75–10.42, *p* < 0.001), and deceased organ donor (cadaveric vs. living, aOR = 2.01, 95% CI = 1.03–3.93, *p* = 0.04) were the three most important risk factors for the development of PTDM. The cumulative survival rate of patients and allografts was higher in patients without PTDM than in those with PTDM *(p* = 0.007 and 0.041, respectively). Concurrent use of calcineurin inhibitors and mammalian target of rapamycin inhibitors (mTORis) decreased the risk of PTDM (tacrolimus vs. tacrolimus with mTORi, aOR = 0.28, 95% CI = 0.14–0.55, *p* < 0.001). Investigating PTDM risk factors before and modifying immunosuppressant regimens after transplantation may effectively prevent PTDM development.

## 1. Introduction

Post-transplant diabetes mellitus (PTDM) is a common and severe complication in patients who receive immunosuppressive agents after kidney transplantation, and this has a major effect on graft and patient outcomes [1,2,3,4]. PTDM is associated with not only increased mortality and morbidity, but also increased rates of cardiovascular diseases and infections, which are the leading causes of death in kidney transplant recipients [5]. The incidence of PTDM is 9.1–45.3% after 1 year [1,3,6,7,8,9], 10.0–30.0% after 3 years [1,4], and 10.2–15.1% after 5 years [10,11]. Similar to type 2 diabetes, PTDM is characterized by insulin resistance, decompensated insulin release, hypertriglyceridemia, obesity, and hypertension [12]. However, risk factors for PTDM differ from those associated with type 2 diabetes. The common risk factors for PTDM include obesity, sedentary lifestyle, other metabolic syndromes associated with obesity, certain viral infections (e.g., hepatitis C virus and cytomegalovirus), and the use of drugs with diabetogenic effects administered with post-transplantation therapy, including corticosteroids and immunosuppressive agents (e.g., tacrolimus, cyclosporine, and mammalian target of rapamycin inhibitors (mTORis)) [13]. Other immunosuppressive drugs, including azathioprine and mycophenolate mofetil (MMF), are not associated with a disruption in glucose metabolism [14]. The effect of immunosuppressive agents on graft and patient survival remains controversial, especially in Asian populations, and the incidence of and risk factors for PTDM in the Taiwanese population are unclear. Here, we aimed to determine the incidence of and risk factors for PTDM in kidney transplant recipients. Furthermore, the prognosis of PTDM was investigated to determine the difference in graft and patient survival rates between patients with PTDM and those without PTDM.

## 2. Patients and Methods

A retrospective cohort study was conducted in a transplantation center in central Taiwan using electronic medical records. Patients with PTDM were defined as those with a fasting blood glucose level of ≥140 mg/dL recorded on at least two consecutive measurements, or those who required the administration of an oral anti-diabetic drug or insulin for ≥3 months, as described in the World Health Organization/American Diabetes Association international consensus guidelines [7,8], recommendations, and future directions on PTDM [15]. Patients with temporary hyperglycemia after transplantation (e.g., administration of anti-diabetic drug or insulin for <3 months or corticosteroid pulse therapy for acute rejection treatment, leading to temporary hyperglycemia) were classified as patients without PTDM. The review board of Taichung Veterans General Hospital approved the study protocol (CE18303B), and the study was carried out in accordance with the Declaration of Helsinki. The need for patient consent was waived owing to the use of a decoded database in the hospital information system.

### 2.1. Study Population

**Figure 1:**
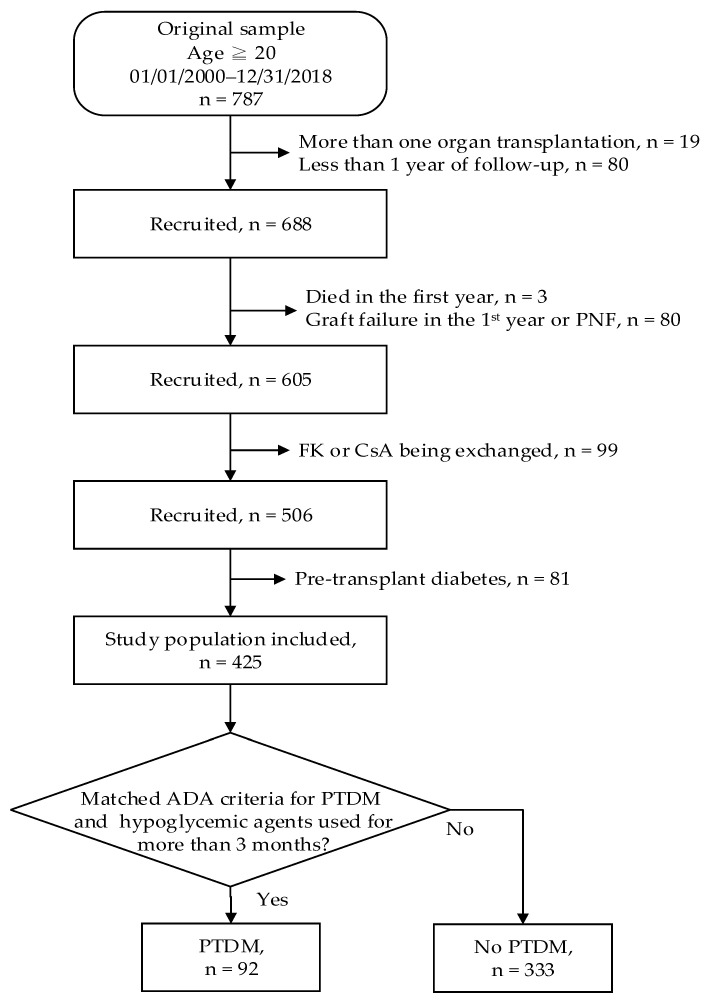
Flowchart of patient inclusion process. ADA: American Diabetes Association; CsA: cyclosporine; FK: tacrolimus; PNF: primary non-function; PTDM: post-transplant diabetes mellitus.

This was a single-center, analytical, retrospective study involving adults (≥20 years old) who underwent a kidney transplant between January 2000 and December 2018 and survived with a functioning allograft for ≥1 year after transplantation. Recipients with more than one organ transplant, post-transplant follow-up of <1 year, graft failure, and/or death in the first year were excluded. Patients with pre-transplant diabetes were also excluded (Figure 1). The primary endpoint of the study was the incidence of and risk factors for PTDM in kidney transplant recipients, and the secondary endpoint was allograft and patient survival. The end time of PTDM risk analyses was the date of PTDM diagnosis or study end.

### 2.2. Immunosuppressant Regimens (IMRs)

Post-transplantation immunosuppressant regimens, including calcineurin inhibitors (CNIs), MMF, and corticosteroids, were used, and mTORis were added as required to prevent organ rejection. Recipients were divided into four groups according to the IMRs received to analyze the correlation and hazards linking IMRs and PTDM: FK group, tacrolimus-based regimen; CsA group, cyclosporine-based regimen; FK + mTORi group, tacrolimus-based regimen combined with an mTORi; and CsA + mTORi group, cyclosporine-based regimen combined with an mTORi. Patients who were initially administered tacrolimus or cyclosporine and whose regimen was altered were excluded.

### 2.3. Statistical Analysis

Continuous variables are expressed as median (interquartile range, IQR). Mann–Whitney U and chi-square tests were used to compare continuous and categorical variables in PTDM-negative and PTDM-positive patients. Survival curves were analyzed using the Kaplan–Meier method. Independent risk factors for PTDM were identified using a stepwise forward multivariate logistic regression model. Statistical analyses were performed using SPSS version 22.0 statistical software (SPSS Company, New York, NY, USA) and *p* < 0.05 indicated statistical significance.

## 3. Results

### 3.1. Study Population

A total of 787 patients underwent renal transplantation, and 425 were enrolled in this study (Figure 1). There were 221 (52.0%) male and 204 (48.0%) female patients (Table 1). The median age of the study population at transplant was 44.0 (IQR = 34.1–51.9) years and the median follow-up period was 10.3 (IQR = 6.4–13.8) years. 

### 3.2. Incidence of PTDM

The median time to PTDM diagnosis was 2.62 (IQR = 0.4–5.56) years. The frequency of PTDM after 10 years was 21.6% (92/425). The incidence rate was the highest in the first year after transplantation (7.7%), followed by the second year (4.0%). The cumulative incidence was 25.6% over 10 years (Table 2). There were no obvious differences with regard to gender or hemodialysis before transplantation between patients in the PTDM and non-PTDM groups (Table 1). The incidence of PTDM was significantly higher in patients with high age and body mass index (BMI; *p* < 0.001). Among the 92 patients who developed PTDM, 75 received renal transplants from deceased donors, demonstrating significant differences in the development of PTDM among patients receiving organs from different donor sources (*p* = 0.004). Hypertension was the most common comorbidity observed in patients receiving kidney transplants in this study, and the frequency of this comorbidity was significantly higher in the non-PTDM group than in the PTDM group (non-PTDM vs. PTDM, 64.0% vs. 47.8%; *p* < 0.007). Infection with cytomegalovirus or hepatitis C virus before transplantation was not significantly related to the development of PTDM. Furthermore, the frequency of recipients of tacrolimus-based IMRs was significantly higher in the PTDM group than in the non-PTDM group (non-PTDM vs. PTDM, 55.0 vs. 69.6%; *p* < 0.001).

### 3.3. Risk Factors of PTDM

**Figure 2:**
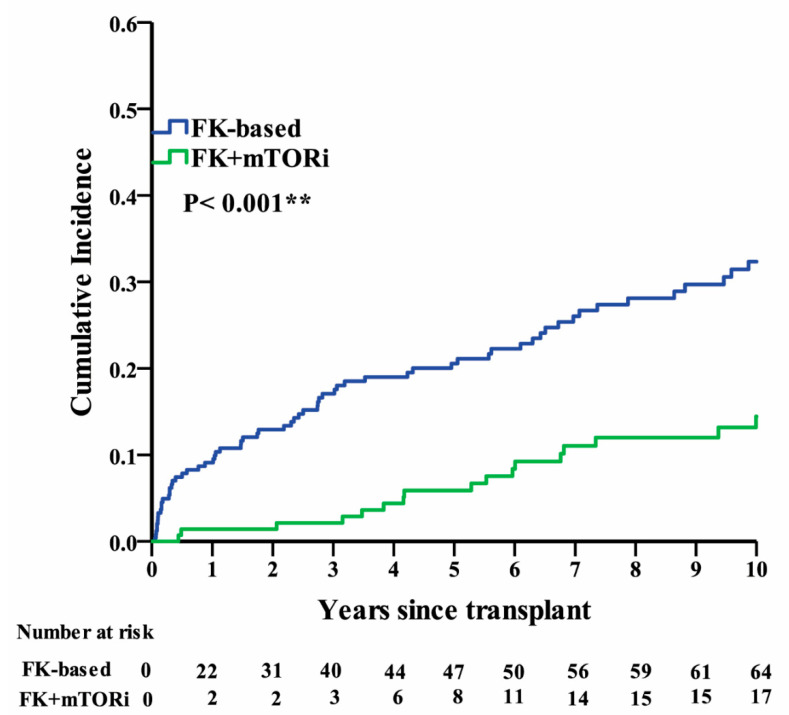
Kaplan–Meier analysis of the cumulative incidence of post-transplant diabetes mellitus (PTDM) in the tacrolimus (FK)-based and FK + mammalian target of rapamycin inhibitor (mTORi)-based regimen groups.

The multivariable logistic regression analyses revealed that age (*p* < 0.001), BMI (*p* < 0.001), donor source (*p* < 0.04), and tacrolimus use (*p* < 0.001) were independent risk factors for PTDM (Table 3). The risk of developing PTDM in recipients aged 45–65 years at the time of transplant was 2.9 times higher than that in patients aged <45 years (aOR = 2.90, 95% confidence interval (CI) = 1.64–5.13), and the risk in patients aged >65 years was 4.86 times higher than that in patients aged <45 years (aOR = 4.86, 95% CI = 1.5–15.79). The risk of developing PTDM in recipients with BMI 24–27 and >27 kg/m^2^ was 2.96 times (aOR = 2.96, 95% CI = 1.52–5.75) and 5.25 times (aOR = 5.25, 95% CI = 2.75–10.42) higher than that in patients with BMI < 24, respectively. The risk of developing PTDM in patients who received cadaveric donor renal transplants was 2.01 times higher than that in patients who received living-patient renal transplants (OR = 2.01, 95% CI = 1.03–3.93). Furthermore, the risk of developing PTDM in recipients of tacrolimus-based IMRs was 3.57 times higher than that in patients receiving the tacrolimus-based + mTORi regimen (OR = 3.57, 95% CI = 0.14–0.55). Finally, the cumulative incidence of PTDM in recipients of tacrolimus-based IMRs was significantly higher than that in patients receiving the tacrolimus-based + mTORi regimen (*p* < 0.001; Kaplan–Meier analysis; Figure 2).

### 3.4. Patient and Allograft Survival rates

**Figure 3:**
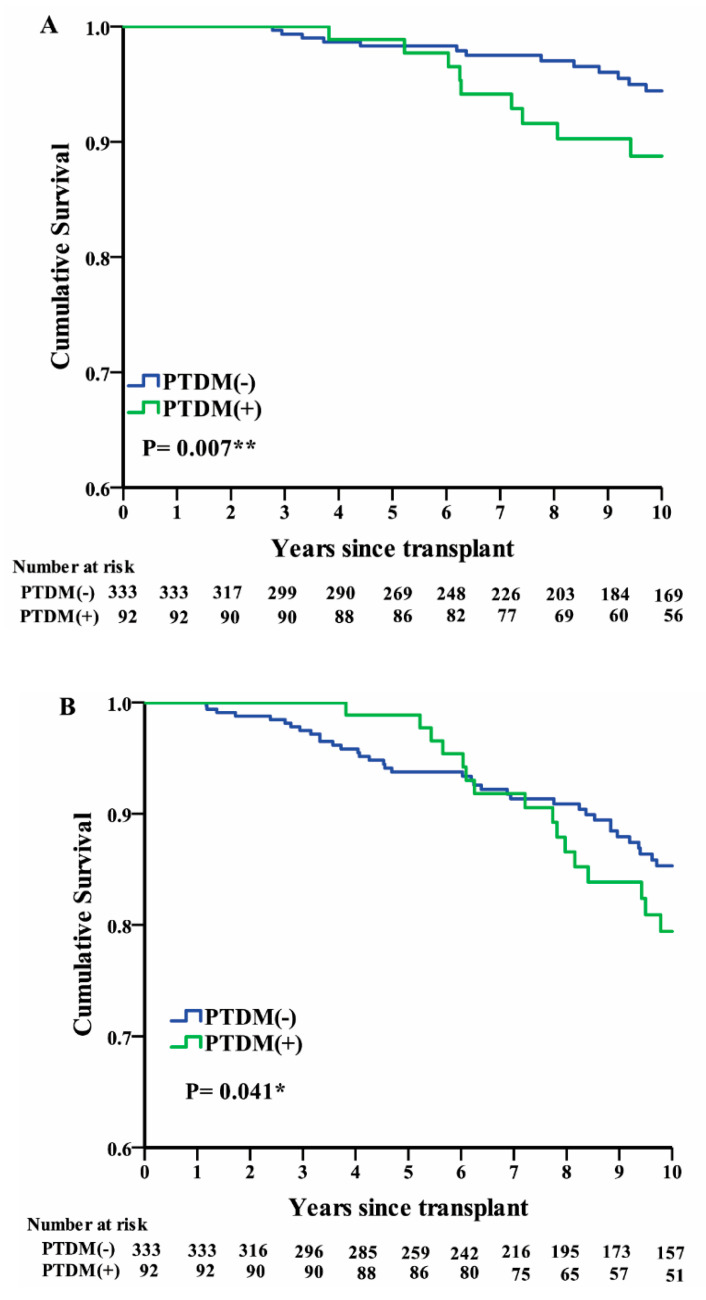
Cumulative survival rates in the post-transplant diabetes mellitus (PTDM) and non-PTDM groups for (**A**) transplant recipients and (**B**) kidney allografts.

A Kaplan–Meier analysis revealed that the 10-year cumulative survival rate of transplant recipients was significantly lower in the PTDM group than in the non-PTDM group (*p* < 0.007; Figure 3A). The 10-year cumulative graft survival rate was also significantly lower in the PTDM group than in the non-PTDM group (Figure 3B).

## 4. Discussion

### 4.1. Incidence of PTDM

We examined the incidence of and risk factors for PTDM after kidney transplantation in Taiwanese patients, and there have been few such studies in the past. At 1, 3, and 5 years after transplantation, the proportion of patients who developed PTDM (Table 2) was not similar to that reported in previous studies (e.g., 13.00% (American study) and 11.80% (Korean study) after 1 year, 30.72% after 3 years (Chinese study), and 15.10% after 5 years (Japanese study)) [2,10,16,17]. The incidence rate of PTDM in both the American and Korean studies was higher than that reported here; this could be attributed to the relatively higher BMI in the American study (27 ± 5 kg/m^2^) or the inclusion of patients with temporary hyperglycemia resulting from the consumption of high doses of corticosteroids to prevent acute rejection in the Korean study [2,16]. The 3-year cumulative incidence of PTDM in the Chinese study was higher than that reported here, and this can be attributed to the inclusion of patients with temporary hyperglycemia in the Chinese study, as in the Korean study [16,17]. However, the 5-year cumulative incidence rate of PTDM in the Japanese study was lower than that in our study, which may be related to the lower serum tacrolimus concentration in the Japanese study (3.8–7.6 ng/mL). The variability in the incidence of PTDM among these studies is due to the diagnostic criteria for PTDM, therapeutic protocols for post-transplant immunosuppression, duration of follow-up, and study population; therefore, the studies may have some deviations in the results. The main purpose of this study was to investigate the long-term incidence of PTDM after kidney transplantation; consequently, patients with temporary PTDM were excluded. Thus, this is a distinguishing feature of our study.

### 4.2. Risk Factors of PTDM

The following four variables with an effect on PTDM development were identified: age (*p* < 0.001), BMI (*p* < 0.001), donor source (*p* < 0.004), and immunosuppressive agent used (*p* = 0.001; Table 1). Using multivariate logistic regression methods, we showed that the risk of developing PTDM in recipients aged ≥45 years was 2.9–4.86 times higher than that in patients aged <45 years, highlighting the correlation between age and PTDM risk [10,16,17,18].

A high BMI in the context of a transplant may affect transplant outcomes, which has been shown in previous studies [3,19,20,21]. Our results showed that BMI was positively correlated with the OR of developing PTDM. There were no patients with obesity in this study; thus, the incidence of PTDM was lower than that reported in other studies.

The donor source was an independent risk factor for PTDM, as recipients of deceased donor kidneys had a 1.5–3.7 times higher risk of developing PTDM than those receiving live donor kidneys [19,22]. The ORs of developing PTDM were higher in patients receiving a deceased donor kidney than in patients receiving a live donor organ (Table 3), and this is similar to results previously reported [19,22]. We postulate that this may be related to the greater use of immunosuppressants (i.e., induction therapy, higher levels of CNI, and higher steroid doses) in deceased donor transplant recipients. Although further evidence is required to support the increased risk of developing PTDM associated with deceased donor transplants, deceased donor transplant recipients should be identified as having a higher risk of developing PTDM than live donor recipients, informed, and cautiously monitored [22].

The use of tacrolimus (vs. cyclosporine) is independently associated with PTDM in several studies [3,22,23]. However, the incidence of PTDM among recipients of tacrolimus and mTORi concurrently (vs. cyclosporine and mTORi) was considerably lower than that among patients receiving CNI-based regimens (Table 1). Our multivariate logistic regression analyses showed that recipients of cyclosporine-based regimens had a higher risk of developing PTDM than those receiving tacrolimus-based regimens, although this was not statistically significant. Therefore, the diabetogenic effects of both cyclosporine and tacrolimus were comparable (Table 2). Recipients concurrently using tacrolimus and mTORis also had a considerably lower risk of developing PTDM than those receiving tacrolimus-based regimens (*p* < 0.001). However, this was in contrast to the results reported by Johnston et al. using the United States Renal Data System registry [24].

The use of CNIs such as cyclosporine and tacrolimus remains the backbone of immunosuppressive treatment for most kidney transplant recipients in our center, and an mTORi may be added to minimize the adverse effects of CNIs and prevent organ rejection. Individualized adjustment of IMRs for kidney transplant recipients helps to maintain graft function and decrease the adverse reactions associated with IMRs, which in turn may protect grafts from the risk of rejection. The diabetogenic effects of tacrolimus are relatively strong and dose-related [23,25]. When tacrolimus is administered with an mTORi to prevent acute rejection, both tacrolimus and mTORi may be used at lower doses; thus, the diabetogenic effects of tacrolimus are reduced [26,27,28]. This may explain why patients receiving concurrent therapy with tacrolimus and mTORi had a lower risk of developing PTDM than those receiving tacrolimus- or cyclosporine-based regimens. In addition, insulin sensitivity does not significantly change when non-obese recipients (BMI < 30 kg/m^2^) are administered tacrolimus or sirolimus [20]. This may also indirectly explain the lower incidence of PTDM observed in the PTDM group in our study, as the median BMI was 24.6 kg/m^2^. A higher prevalence of hypertension was observed in the non-PTDM group than in the PTDM group (Table 1). A meta-analysis study reported that renin-angiotensin system (RAS) inhibitors (angiotensin-converting enzyme inhibitors or angiotensin receptor blockers) reduced the incidence of newly diagnosed diabetes (27% and 23%, respectively) [29]. Investigation on the use of RAS in our study population showed that patients in the non-PTDM group used more RAS inhibitors than patients in the PTDM group (48.6% vs. 25.0%, *p* < 0.001). Therefore, it is speculated that the long-term use of RAS inhibitors may protect patients with hypertension before transplantation from PTDM risk. This may also explain why patients with high blood pressure before transplantation have a lower risk of PTDM incidence.

### 4.3. Patient and Allograft Survival Rates

Although studies had shown that the major causes of death in patients with PTDM are related to cardiovascular disease [30,31], the use of immunosuppressants after kidney transplantation can allow severe infection and even death in patients with PTDM [32]. In our study, infection was the main cause of death after transplantation (Table 1), which may explain the lower survival rates after 10 years in patients with PTDM than in patients without PTDM (Figure 3A). Thus, the increased mortality risk in patients with PTDM may have been related to drug–drug interactions and infections associated with immunosuppressant medications. In our study, the allograft survival rate after 10 years was significantly lower in recipients in the PTDM group, which was in accordance with evidence published by Kuo et al. using the Organ Procurement and Transplant Network/United Network for Organ Sharing database [33].

### 4.4. Study Limitations

This study had certain limitations. First, the study population only represented patients receiving kidney transplantations in a single center, indicating that these results should be generalized with caution. Second, tacrolimus was made available in 1998 and became popular because of its effects in preventing acute rejection. Cyclosporine is less frequently prescribed and statistical errors may have occurred because of the differences in patient sample sizes. Finally, only patients who underwent just one transplantation were included for analysis in the study, and those whose treatment was switched between tacrolimus and cyclosporine were excluded (*n* = 99). Thus, it is difficult to extrapolate the frequency of PTDM in patients who received multiple transplants and the correlation between PTDM and changes in tacrolimus/cyclosporine prescription.

## 5. Conclusions

This study revealed that high age and BMI at transplantation and receiving an organ from a deceased donor were the three most important risk factors for developing PTDM. The cumulative survival rates of patients and allografts were higher in patients with PTDM than in those without PTDM. Furthermore, concurrent use of CNIs and mTORis may decrease the risk of PTDM. Screening patients to identify PTDM risk factors before transplantation and modifying immunosuppressant regimens after transplantation may effectively prevent the development of PTDM. Further prospective research is needed to understand the relationship between immunosuppressant regimens and PTDM. 

## Figures and Tables

**Table 1:** Characteristics of the kidney transplantation study population.

Variable	Non-PTDM (*n* = 333)		PTDM (*n* = 92)		*p*-Value
Age at KT, years †	41.8	(33.0–50.8)	49.7	(40.5–56.9)	<0.001 **
Gender					0.272
Female	165	(49.5%)	39	(42.4%)	
Male	168	(50.5%)	53	(57.6%)	
BMI †	22.3	(20.2–24.6)	24.6	(22.6–27.7)	<0.001 **
Donor source					0.004 **
Living	114	(34.2%)	17	(18.5%)	
Cadaveric	219	(65.8%)	75	(81.5%)	
Dialysis pre-transplant					1.000
HD/PD	283	(85.2%)	78	(84.4%)	
pre-empty	49	(14.8%)	14	(15.2%)	
Causes of patient death					0.036 *
Infection	8	(40.0%)	9	(60.0%)	
Cancer	11	(55.0%)	1	(6.7%)	
Hepatic failure	0	(0.0%)	2	(13.3%)	
Cardiovascular sudden death	0	(0.0%)	1	(6.7%)	
CVA	0	(0.0%)	1	(6.7%)	
Others	1	(5.0%)	1	(6.7%)	
Comorbidity before KT					
Hypertension	213	(64.0%)	44	(47.8%)	0.007 **
Anemia	154	(46.2%)	37	(40.2%)	0.362
Hyperuricemia	82	(24.6%)	14	(15.2%)	0.077
Hyperlipidemia	75	(22.5%)	17	(18.5%)	0.490
Hepatitis B	16	(4.8%)	3	(3.3%)	0.776
Hepatitis C	13	(3.9%)	2	(2.2%)	0.540
Cancer	8	(2.4%)	3	(3.3%)	0.710
MACE	2	(0.6%)	0	(0.0%)	1.000
CMV	3	(0.9%)	1	(1.1%)	1.000
BK viremia	2	(0.6%)	0	(0.0%)	1.000
IMRS groups					0.001 **
FK	183	(55.0%)	64	(69.6%)	
FK + mTORi	124	(37.2%)	17	(18.5%)	
CsA	12	(3.6%)	9	(9.8%)	
CsA + mTORi	14	(4.2%)	2	(2.2%)	

Chi-square test. † Mann–Whitney U test. * *p* < 0.05, ** *p* < 0.01. PTDM: post-transplant diabetes mellitus; BMI: body mass index; CMV: cytomegalovirus; CVA: cerebrovascular accident; KT: kidney transplantation; HD/PD: hemodialysis/peritoneal dialysis; IMRs: immunosuppressive regimens; FK: tacrolimus; MACE: major adverse cardiovascular events; mTORi: mammalian target of rapamycin inhibitor; CsA: cyclosporine.

**Table 2:** Incidence of PTDM during the follow-up periods (*n* = 425).

Years post KT	1	2	3	4	5	6	7	8	9	10
Total patients	388	362	335	315	290	262	233	206	185	166
Patients with PTDM	30	10	10	10	6	6	9	4	2	5
Incidence (%)	7.73	2.76	2.99	3.17	2.07	2.29	3.86	1.94	1.08	3.01
Cumulative patients	30	40	50	60	66	72	81	85	87	92
Cumulative incidence (%)	7.15	9.58	12.13	14.78	16.44	18.24	21.18	22.59	23.39	25.57

KT: kidney transplantation; PTDM: post-transplant diabetes mellitus.

**Table 3:** Logistic regression analysis for risk factors for PTDM.

	Univariable		Multivariable	
	OR (95% CI)	*p*-Value	OR (95% CI)	*p*-Value
Gender (vs. female)				
Male	1.33 (0.84–2.13)	0.23	1.30 (0.76–2.21)	0.33
Age at KT, years (vs. <45 years)				
45–65	2.62 (1.60–4.29)	<0.001 **	2.90 (1.64–5.13)	<0.001 **
>65	3.38 (1.17–9.80)	0.03 *	4.86 (1.50–15.79)	0.008^**^
Donor source (vs. living)				
Cadaveric	2.30 (1.29–4.07)	0.004 **	2.01 (1.03–3.93)	0.040 *
BMI, kg/m^2^ (vs. BMI <24)				
24–27	2.75 (1.53–4.92)	0.001 **	2.96 (1.52–5.75)	0.001 **
>27	4.15 (2.32–7.44)	<0.001 **	5.35 (2.75–10.42)	<0.001 **
IMR groups (vs. FK-based)				
FK + mTORi	0.39 (0.22–0.70)	0.002 **	0.28 (0.14–0.55)	<0.001 **
CsA	2.14 (0.86–5.33)	0.10	1.40 (0.49–3.97)	0.53
CsA + mTORi	0.41 (0.09–1.85)	0.25	0.30 (0.06–1.64)	0.17

* *p* < 0.05, ** *p* < 0.01. PTDM: post-transplant diabetes mellitus; BMI: body mass index; KT: kidney transplantation; IMR: immunosuppressive regimen; FK: tacrolimus; mTORi: mammalian target of rapamycin inhibitor; CsA: cyclosporine.
